# Referral patterns to primary mental health services in Western Sydney (Australia): an analysis of routinely collected data (2005–2018)

**DOI:** 10.1186/s13033-020-00368-5

**Published:** 2020-05-26

**Authors:** Sithum Munasinghe, Andrew Page, Haider Mannan, Shahana Ferdousi, Brendan Peek

**Affiliations:** 1 Translational Health Research Institute, Western Sydney University, Campbelltown Campus, Locked Bag 1797, Penrith, NSW 2751 Australia; 2 Western Sydney Primary Health Network, Level 1/85 Flushcombe Road, Blacktown, NSW 2148 Australia; 3 Royal Australasian College of Dental Surgeons, Level 13, 37 York Street, Sydney, NSW Australia

**Keywords:** Primary mental health care services, Access to Allied Psychological Services, Referral, Western Sydney

## Abstract

**Background:**

Regionally-specific approaches to primary mental health service provision through Primary Health Networks (PHNs) have been a feature of recent national mental health reforms. No previous studies have been conducted to investigate local patterns of primary mental health care (PMHC) services in Western Sydney. This study is designed to (i) understand the socio-demographic and economic profiles (ii) examine the inequalities of service access, and (iii) investigate the service utilisation patterns, among those referred to PMHC services in Western Sydney, Australia.

**Methods:**

This study used routinely collected PMHC data (2005–2018), population-level general practice and Medicare rebates data (2013–2018) related to mental health conditions, for the population catchment of the Western Sydney PHN. Sex- and age-specific PMHC referrals were examined by socio-demographic, diagnostic, referral- and service-level factors, and age-specific referrals to PMHC services as a percentage of total mental health encounters were investigated.

**Results:**

There were 27,897 referrals received for 20,507 clients, of which, 79.19% referrals resulted in follow-up services with 138,154 sessions. Overall, 60.09% clients were female, and median age was 31 years with interquartile ranged 16–46 years. Anxiety and depression were the predominant mental health condition, and 9.88% referred for suicidal risk. Over two-thirds of referrals started treatments during the first month of the referral and 95.1% of the total sessions were delivered by face to face. The younger age group (0–24) had greater referral opportunities as a percentage of total visits to a general practitioner and Medicare rebates, however demonstrating poor attendance rates with reduced average sessions per referral compared with older adults.

**Conclusion:**

Children and young adults were more likely to be referred to PMHC services than older adults, but were less likely to attend services. Further research is needed to identify the strategies to address these differences in access to PMHC services to optimise the effectiveness of services.

## Introduction

Mental and substance use disorders are the second leading cause of disease burden in Australia, accounting for 15% of total disease burden in terms of disability adjusted life years [1]. The most recent National survey of mental health and wellbeing conducted in 2007 suggested that up to one in every five Australians experienced a mental disorder in the previous 12-months, with anxiety and depressive disorders being the most common [2]. However, access and utilisation of mental health services for those with a mental disorder has not corresponded with the underlying disease burden in the population, in both the most recent [2] and earlier national mental health surveys [3], with approximately two-thirds of those with a mental disorder not accessing a mental health service. Factors affecting access and utilisation to mental health historically have related to financial barriers, geographic isolation and service capacity in rural and remote locations, and the stigma associated with mental disorder [2, 4]. Additionally, differential access is evident among specific socio-demographic groups, for example children and adolescents, people from culturally and linguistically diverse backgrounds, and Aboriginal and Torres Strait Islander populations [5–7].

In response to this significant population health burden, the federal government established the Better Outcomes in Mental Health Care Programme in 2001 to facilitate access to mental health services. As part of this program, General Practitioners (GPs) were provided incentives to work collaboratively with mental health professionals under the Access To Allied Psychological Services (ATAPS) and to refer patients with mental disorders to receive psychological therapies at no cost or at low cost [8]. These programs have been widened through several initiatives; headspace -established in 2006, which delivers early intervention to youth aged 12–24 experiencing or at risk of mental health conditions [9]; and, Better Access—which is Australia’s universal mental health initiative established in 2007 [10], during the last two decades. Recent figures indicate the Commonwealth has invested approximately $9 billion across all primary and acute mental health services in 2015–2016 [11]. However, despite these wider investments in mental health services, the burden associated with mental disorders has not changed substantially [12].

A major review of mental health services conducted in 2014 by the National Mental Health Commission suggested that primary and acute care mental health services were not adequately addressing the burden associated with mental health disorders. Recommendations from this review suggested a more regional approach to mental health and suicide prevention services, administered by Primary Health Networks (PHNs), and a greater focus on articulating pathways from primary, secondary, and tertiary mental health services [13].

This new form of access to primary mental health care (PMHC) services has provided an opportunity to design and arrange locally relevant interventions, treatment options and establish new policies and guidelines by acknowledging regional heterogeneity in terms of socio-demographics and service needs in order to optimize the cost-effectiveness of substantial investments. However, this presents challenges to local decision makers and it requires; (i) an understanding of the sociodemographic and socioeconomic profiles among those accessing PMHC services; (ii) to determine whether potential inequalities in referrals to PMHC services; and (iii) an understanding of subsequent service utilisation patterns among those referred to the PMHC services, in the period prior, during, and subsequent to these recent local policy changes. Accordingly, this study aims to investigate referral pathways and service utilization patterns of PMHC services in the Western Sydney region of Australia.

## Methods

This study was based on all individuals referred to key PMHC services (see below) over the period 2005–2018 in the geographic catchment of Western Sydney PHN, covers four Local Government Areas including Blacktown, Parramatta, Cumberland and the Hills Shire in the greater Western Sydney region. The population of the study catchment area was approximately one million people in 2018 [14], with 344 general practices in 2017 [15].

### Primary mental health care services

PMHC services considered in this study included federal government funded mental health initiatives established to provide access to mental health treatments to those who otherwise have low or no access to mental health services. These services are currently focus on six main service areas including (i). Low intensity psychological interventions to those with mild mental health conditions, (ii). Psychological therapies delivered by mental professionals, (iii). Early interventions for youth and children, (iv). Treatments to those with severe and complex mental health conditions, (v). Indigenous specific mental health conditions and (vi). Suicide prevention services.

A range of professionals can refer clients to the PMHC services including general practitioners, allied mental health professionals, education providers, social workers and other professionals. Clients can also self-refer. All clients generally remain in contact with their GP, including those who self-refer to PMHC services. Prior to the commencement of treatments, an initial assessment is required to decide the appropriate level of care. The initial assessment may be undertaken by a general practitioner or referring clinician, hospital intake teams, of other community mental health services commissioned by the PHN. Following an initial assessment, usually provided by a general practitioner or other clinician, individuals are referred to an appropriate level of care ranging from low intensity to severe or complex care, and the initial diagnosis may change during the course of follow-up treatments. Progress is monitored during the treatment period, to allow modifications to the type and intensity of treatments [16].

### Data sources

Data for key PMHC services were extracted from two main data sources: (i) the national PMHC minimum data set (MDS) for the period 2016 to 2018 and (ii) the national ATAPS MDS for the period 2005 to 2018. The PMHC MDS comprises items relating to patients’ socioeconomic and demographic information, mental health diagnosis information, service session information and practitioner characteristics. The PMHC MDS was developed based on the previous ATAPS MDS data architecture, but collects a broader range of information relevant to all PMHC services. Since the establishment of the PMHC MDS in July 2016, all new ATAPS encounters information were subsumed within this dataset in July 2017 and ATAPS MDS was suspended in August 2018. The generic term ‘PMHC’ is used in the current study to refer to mental health services which focus on the six main treatment areas across both these datasets.

In addition to PMHC data, the current study also used general practice data relating to mental health conditions for the three most recent calendar year period from 2015 July to 2018 June from an internal database held at Western Sydney PHN. This database routinely collects data from 202 general practices out of 344 in the Western Sydney PHN population catchment and provides total numbers of patients who visited a GP. This database has categorized presenting diagnoses into major diagnosis groups, and the current study extracted patients for mental health related diagnoses coded as: anxiety, attention deficit hyperactivity disorder, bipolar disorders, depression, postnatal depression and schizophrenia. In addition, number of patients received Medicare rebates related to mental health conditions according to Medicare Benefits Schedule (MBS) items for the Western Sydney PHN catchment was extracted from a publicly accessible source which contains data for five calendar years from 2012 July to 2017 June [17]. Both the number of GP visits and MBS services related to mental health conditions were used as denominators for PMHC referrals in the subsequent analyses.

Population denominators for the Western Sydney PHN catchment were also obtained from Australian Bureau of Statistics population data [14]. Additionally, the socio-economic status of the area of residence for each individual was defined based on the Index of Relative Socioeconomic Advantage and Disadvantage (IRSAD) of suburbs in the Western Sydney PHN catchment, obtained for the Census years 2006, 2011 and 2016 [18] with intercensal IRSAD values calculated by weighted interpolation using the formula IRSAD_i = IRSAD_a + (IRSAD_b − IRSAD_a)/(b − a)*(i − a), for a given year “i” between 2 years “a” and “b”.

### Study factors

A series of key socio-demographic, diagnostic, referral- and service-level factors were defined based on available data in the PMHC data sources described above. Socio-demographic factors included gender (male, female), age (≤11, 12–24, 25–34, 35–44, 45–64, ≥65), and socioeconomic status defined as an area level measure in terms of IRSAD which is divided into five quintiles ranging from most deprived to least deprived.

Diagnostic factors included principal and/or additional diagnosis and suicidal referral status. Diagnosis is made by the referring clinician, and a set of diagnosis categories are then coded in the PMHC MDS according to Diagnostic and Statistical Manual of Mental Disorders IV criteria. In the current study four main categories were defined reflecting high prevalence mental disorders (anxiety disorders, depressive disorders, substance use disorders, other). Suicide risk (yes, no) was defined as ‘yes’ if the referral coming from a hospital or emergency department due to an attempt of intentional self-harm, or presentation to a GP following an attempt of intentional self-harm or with strong suicide ideation.

Mental health referral-level factors included the principal focus of treatment (low intensity psychological intervention, child and youth specific interventions, psychological therapy, other), profession of the referrer (self-referral, paediatrician, allied mental health professionals, GP, psychiatrist, other), and any psychotropic medication use during the presentation (antipsychotics, anxiolytics, hypnotics and sedatives, antidepressants, and psychostimulants and nootropics).

Service-level and other factors included waiting time to receive mental health treatment defined as the interval between the date of referral and the date of first treatment session, the number of sessions per referral, mode of service contact (face to face, telephone, internet based, video), treatment attendance status (attended, not-attended) which is the status of treatment commencement, and the treatment no-show status (yes, no) which is the status of client’s attendance for any scheduled session for those who have already commenced treatment or have agreed to attend treatment.

### Data analysis

Descriptive analyses were conducted to investigate the distribution of presentations, stratified by sex and age, for socio-demographic, diagnostic, referral- and service-level factors. This information was presented as mean and standard error (SE) or median and interquartile range (IQR), where appropriate, for continuous variables, and counts and percentages for categorical variables. Age specific referrals and rates as a percentage of total GP visits and MBS items related to mental health conditions were presented for the most recent five calendar year period from 2013–2014 to 2017–2018. Since some individuals have more than one episodes of care over the 14-year period and information other than gender may be time dependent, referral instead of individual was considered as the unit of the analysis. Observed GP visits relating to mental health conditions were multiplied by 344/202 in order to estimate total GP visits relating to mental health conditions for the total population residing in the Western Sydney PHN catchment. Population counts were assumed to be uniformly distributed between two age values to estimate equivalent age-groups where there were inconsistencies between age-groups between Census population and MBS data sources. Data analysis was conducted using Stata14.0 (Stata Crop, 4905 Lakeway Dive, College Station, Texas 77845, USA).

## Results

**Fig. 1:**
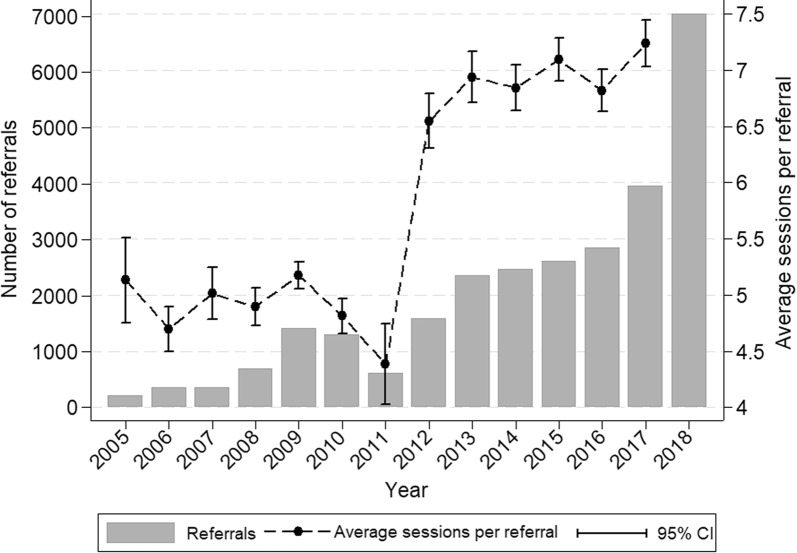
Trends of referrals and sessions per referral for PMHC services, Western Sydney (Australia), 2005–2018. Average sessions per referral has not been calculated for 2018 as not all referrals finished treatment sessions for 2018; *CI* confidence intervals

There were 27,897 referrals to PMHC services over the period 2005–2018 for 20,507 individual clients, with a higher proportion of referrals among females than males (60.1%) (Table 1). Approximately 79% of referrals resulted in a subsequent attendance to a mental health service representing a 138,154 total sessions (Median number of session = 6, interquartile range 3 to 8), with just over 75% of follow-up appointments occurring within 1-month, and the majority of treatments (95%) were delivered by face to face, while clients did not attend for 1971 (1.41%) scheduled sessions (Table 1). Among those referred over the study period, approximately one in ten were presented with suicidal risk (Table 1). Both the number of referrals and the mean number of sessions attended increased over time, rising exponentially in the most recent period (Fig. 1).

**Table 1:** Referrals among those presenting to PMHC services by sex, Western Sydney (Australia), 2005–2018

Characteristics	Male (N = 11,021)	Female (N = 16,597)	Total (N = 27,897)^#^
Age; median (IQR)	26 (11,43)	33 (19,47)	31 (16,46)
Socioeconomic status (IRSAD) (data missing for 2054 referrals)			
1 (most deprived)	2280 (22.15%)	3089 (20.18%)	5422 (20.98%)
2	876 (8.51%)	1287 (8.41%)	2193 (8.49%)
3	2282 (22.17%)	3245 (21.19%)	5582 (21.6%)
4	2423 (23.54%)	3904 (25.5%)	6372 (24.66%)
5 least deprived	2434 (23.64%)	3786 (24.73%)	6274 (24.28%)
Diagnosis (data missing for 2942 referrals)			
Anxiety and affective disorders	2822 (28.78%)	6002 (40.18%)	8901 (35.67%)
Affective (Mood) disorders	2101 (21.43%)	3731 (24.97%)	5897 (23.63%)
Anxiety disorders	2080 (21.21%)	2868 (19.2%)	4977 (19.94%)
Substance use disorders	654 (6.67%)	427 (2.86%)	1088 (4.36%)
Other	2149 (21.92%)	1911 (12.79%)	4092 (16.4%)
Referrer type (data missing for 1568 referrals)			
N/A—self referral	268 (2.57%)	609 (3.89%)	880 (3.34%)
Paediatrician	353 (3.38%)	159 (1.02%)	512 (1.94%)
Allied mental health professionals	470 (4.5%)	416 (2.66%)	889 (3.38%)
Psychiatrist	35 (0.34%)	61 (0.39%)	96 (0.36%)
General Practitioner	9059 (86.81%)	14,046 (89.75%)	23,340 (88.65%)
Other	251 (2.41%)	359 (2.29%)	612 (2.32%)
Principal focus of the treatment (data missing for 60 referrals)			
Low intensity intervention	552 (5.02%)	1279 (7.73%)	1860 (6.68%)
Child and youth-specific	2218 (20.15%)	1598 (9.65%)	3835 (13.78%)
Psychological therapy	7317 (66.48%)	11,743 (70.94%)	19,269 (69.22%)
Other	919 (8.35%)	1933 (11.68%)	2873 (10.32%)
Suicide referral status (data missing for 1695 referrals)^a^			
Yes	1011 (10.12%)	1459 (9.83%)	2473 (9.88%)
No	8979 (89.88%)	13,377 (90.17%)	22,570 (90.12%)
Any mental health medication used (data missing for 4443 referrals)			
Yes	3051 (32.58%)	5448 (39.27%)	8584 (36.6%)
No	6313 (67.42%)	8424 (60.73%)	14,870 (63.4%)
Referrals resulted in services (157 referrals in the waiting list)			
Yes	8628 (78.77%)	13,160 (79.71%)	21,968 (79.19%)
No	2326 (21.23%)	3349 (20.29%)	5772 (20.81%)
Treatment no-show			
Yes	680 (1.25%)	1285 (1.51%)	1971 (1.40%)
No	53,695 (98.75%)	83,659 (98.49%)	138,154 (98.60%)
Waiting time (data missing for 130 referrals)^b^			
Within 7 days	2987 (34.9%)	4777 (36.46%)	7838 (35.89%)
1 week–1 month	3582 (41.85%)	5445 (41.56%)	9075 (41.56%)
1–3 month	1600 (18.69%)	2286 (17.45%)	3919 (17.95%)
3–6 month	286 (3.34%)	432 (3.3%)	734 (3.36%)
> 6 month	104 (1.22%)	161 (1.23%)	272 (1.25%)
Mode of contact (data missing for 12,096 sessions)^c^			
Face to face	46,952 (95.24%)	72,499 (95.14%)	119,929 (95.14%)
Telephone	1946 (3.95%)	3157 (4.14%)	5177 (4.11%)
Internet-based	381 (0.77%)	535 (0.7%)	924 (0.73%)
Video	20 (0.04%)	8 (0.01%)	28 (0.02%)
Session per referral median (IQR)^d^	6 (3,7)	6 (3,8)	6 (3,8)

^#^The sum of males and female counts do not equal the total as gender was missing for 279 referrals; ^a^Since this service was added in 2008, denominator was considered as total referrals from 2008 to 2018; ^b^Referrals not resulted in services excluded from the denominator; ^c^number of sessions were used as the denominator; ^d^Referrals did not finish treatments were not included in the calculation; Amount of missing excluded from the denominator when calculating percentages

There were few differences between males and females across socio-demographic, referral and session level factors (Table 1). Males had a lower median age than females (Male median age = 26, compared to Female median age = 33). Affective and anxiety disorders were the most common condition for referral among females, and males less likely to be diagnosed with an affective or anxiety disorder, but more likely to be diagnosed with a substance use disorder or other mental health diagnoses (Table 1). Males were also more likely to be referred by allied mental health professionals (Table 1).

By contrast, a number of differences were observed across age groups (Table 2). Younger age groups (0–24 years) were more likely to reside in socioeconomically disadvantaged locations, and presented for anxiety disorders, while less likely to receive any mental health medication, compared with older adults (Table 2). Over 75% of referrals attended the first treatment session within the first month, while children aged ≤ 11 waited a considerably longer time to attend the first treatment session, compared with other age groups (Table 2).

**Table 2:** Referrals among those presenting to PMHC services by age-group, Western Sydney (Australia), 2005–2018

Characteristics	Age							
	Median (IQR)	≤ 11 N (%)	12–24 N (%)	25–34 N (%)	35–44 N (%)	45–54 N (%)	55–64 N (%)	≥ 65 N (%)
Total (data missing for 2983 referrals)	31 (16,46)	4280 (17.2)	5653 (22.7)	4122 (16.5)	4054 (16.3)	3391 (13.6)	2124 (8.5)	1290 (5.2)
Gender (data missing for 206 referrals)								
Male	26 (11,43)	2596 (61.1)	2196 (39.2)	1336 (32.7)	1439 (35.8)	1157 (34.4)	756 (35.9)	416 (32.4)
Female	33 (19,47)	1654 (38.9)	3402 (60.8)	2750 (67.3)	2584 (64.2)	2203 (65.6)	1351 (64.1)	868 (67.6)
Socioeconomic status (IRSAD) (data missing for 1664 referrals)								
1 (Most deprived)	28 (13,44)	1119 (27.1)	1110 (21.1)	819 (21.7)	761 (20.5)	584 (18.6)	410 (20.6)	216 (17.6)
2	33 (20,47)	287 (6.9)	372 (7.1)	393 (10.4)	345 (9.3)	303 (9.6)	219 (11.0)	75 (6.1)
3	29 (14,44)	964 (23.3)	1187 (22.5)	794 (21.0)	762 (20.5)	586 (18.6)	369 (18.5)	279 (22.7)
4	33 (18,49)	834 (20.2)	1217 (23.1)	900 (23.8)	940 (25.3)	819 (26.1)	596 (29.9)	352 (28.7)
5 least deprived	30 (16,46)	926 (22.4)	1385 (26.3)	870 (23.0)	901 (24.3)	851 (27.1)	400 (20.1)	305 (24.9)
Diagnosis (data missing for 2589 referrals)								
Anxiety and affective disorders	36 (24,49)	340 (9.2)	1849 (35.5)	1671 (45.2)	1575 (43.4)	1375 (46.0)	854 (45.4)	496 (41.3)
Affective (mood) disorders	36 (22,50)	325 (8.7)	1190 (22.8)	941 (25.4)	972 (26.8)	853 (28.6)	587 (31.2)	396 (32.9)
Anxiety disorders	17 (10,37)	1471 (39.6)	1228 (23.6)	500 (13.5)	466 (12.8)	329 (11.0)	241 (12.8)	206 (17.1)
Substance use disorders	36 (27,45)	19 (0.5)	177 (3.4)	233 (6.3)	267 (7.4)	168 (5.6)	71 (3.8)	27 (2.2)
Other	13 (8,33)	1560 (42.0)	764 (14.7)	355 (9.6)	351 (9.7)	261 (8.7)	130 (6.9)	77 (6.4)
Referrer type (data missing for 1434 referrals)								
N/A—Self referral	39 (31,49)	3 (0.1)	46 (0.8)	91 (2.4)	123 (3.3)	97 (3.2)	34 (1.8)	15 (1.2)
Paediatrician	8 (6,10)	419 (9.8)	93 (1.7)	0 (0)	0 (0)	0 (0)	0 (0)	0 (0)
Allied mental health professionals	12 (9,26)	442 (10.4)	211 (3.9)	81 (2.1)	53 (1.4)	50 (1.6)	35 (1.8)	13 (1.1)
Psychiatrist	34 (18,46)	7 (0.2)	24 (0.4)	19 (0.5)	16 (0.4)	19 (0.6)	9 (0.5)	1 (0.1)
General practitioner	31 (16,46)	3302 (77.4)	4963 (90.7)	3514 (92.5)	3464 (92.7)	2841 (92.7)	1803 (94.3)	1153 (93.4)
Other	29 (15,45)	92 (2.2)	133 (2.4)	93 (2.4)	81 (2.2)	58 (1.9)	30 (1.6)	52 (4.2)
Principal focus of the treatment (data missing for 59 referrals)								
Low intensity intervention	41 (29,53)	27 (0.6)	143 (2.5)	245 (6.0)	262 (6.5)	240 (7.1)	136 (6.4)	91 (7.1)
Child and youth–specific	9 (7,12)	2738 (64.0)	1031 (18.2)	0 (0)	0 (0)	0 (0)	0 (0)	0 (0)
Psychological therapy	35 (21,49)	1466 (34.3)	3842 (68.0)	3049 (74.3)	3179 (78.9)	2706 (79.9)	1756 (82.8)	1113 (86.5)
Other	33 (25,45)	45 (1.1)	637 (11.3)	811 (19.8)	587 (14.6)	439 (13)	230 (10.8)	82 (6.4)
Suicide referral status (data missing for 1236 referrals)^a^								
Yes	33 (22,46)	36 (0.9)	721 (13.3)	556 (14.6)	452 (12.2)	399 (13.0)	198 (10.4)	75 (6.2)
No	30 (14,45)	4148 (99.1)	4703 (86.7)	3258 (85.4)	3260 (87.8)	2669 (87.0)	1699 (89.6)	1137 (93.8)
Any mental health medication used (data missing for 3566 referrals)								
Yes	39 (26,51)	282 (7.3)	1377 (27.5)	1520 (43.9)	1593 (47.5)	1505 (53.3)	945 (55.1)	555 (50.3)
No	22 (11,40)	3590 (92.7)	3637 (72.5)	1944 (56.1)	1763 (52.5)	1318 (46.7)	770 (44.9)	549 (49.7)
Referrals resulted in services (157 referrals in the waiting list)								
Yes	32 (16,47)	3316 (77.8)	4241 (75.7)	3148 (76.7)	3206 (79.6)	2818 (83.6)	1811 (85.8)	1099 (86.0)
No	27 (14,41)	948 (22.2)	1363 (24.3)	955 (23.3)	823 (20.4)	551 (16.4)	299 (14.2)	179 (14.0)
Treatment no–show								
Yes	NA	219 (1.1)	495 (1.8)	371 (1.8)	320 (1.4)	258 (1.3)	135 (1.0)	79 (1.0)
No	NA	19,739 (98.9)	26,963 (98.2)	20,052 (98.2)	21,938 (98.6)	19,739 (98.7)	13,569 (99.0)	7610 (99.0)
Waiting time (data missing for 105 referrals)^b^								
within 7 days	35 (21,49)	611 (18.6)	1433 (34.0)	1266 (40.4)	1215 (38.1)	1122 (39.9)	706 (39.1)	388 (35.5)
1 week–1 month	31 (15,47)	1580 (48.0)	1816 (43.1)	1259 (40.2)	1336 (41.9)	1114 (39.7)	745 (41.3)	495 (45.2)
1–3 month	26 (12,45)	882 (26.8)	793 (18.8)	489 (15.6)	474 (14.9)	460 (16.4)	284 (15.7)	164 (15.0)
3–6 month	29 (12,44)	159 (4.8)	131 (3.1)	84 (2.7)	116 (3.6)	78 (2.8)	50 (2.8)	33 (3.0)
> 6 month	31 (12,46)	60 (1.8)	37 (0.9)	37 (1.2)	48 (1.5)	35 (1.2)	20 (1.1)	14 (1.3)
Mode of contact (data missing for 9324 sessions)^c^								
Face to face	NA	18,357 (99.0)	23,862 (96.3)	17,155 (93.8)	18,992 (94.5)	17,110 (93.9)	12,300 (94.5)	7217 (97.8)
Telephone	NA	165 (0.9)	650 (2.6)	920 (5.0)	937 (4.7)	927 (5.1)	594 (4.6)	148 (2.0)
Internet–based	NA	12 (0.1)	254 (1.0)	208 (1.1)	151 (0.8)	166 (0.9)	118 (0.9)	15 (0.2)
Video	NA	0 (0)	1 (0)	7 (0)	9 (0)	10 (0.1)	1 (0)	0 (0)
Session per referral^d^	NA	6 (3,7)	6 (3,8)	6 (3,8)	6 (3,9)	6 (3,10)	6 (3,11)	6 (3,9)

^a^Since this service was added in 2008, denominator was considered as total referrals from 2008 to 2018; ^b^Referrals not resulted in services excluded from the denominator; ^c^number of sessions were used as the denominator; ^d^Referrals did not finish treatments were not included in the calculation; Amount of missing excluded from the denominator when calculating percentages

**Fig. 2:**
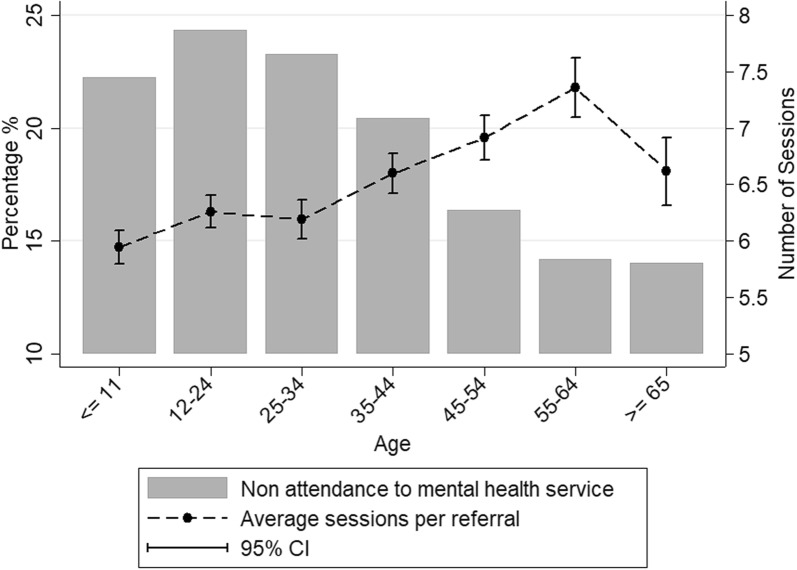
Non-attendance to PMHC services and sessions per referral by age-group, Western Sydney (Australia), 2005–2018. Number of session in 2018 were excluded from the calculation of average sessions per referral as not all referrals finished treatment sessions for 2018; *CI* confidence interval

**Fig. 3:**
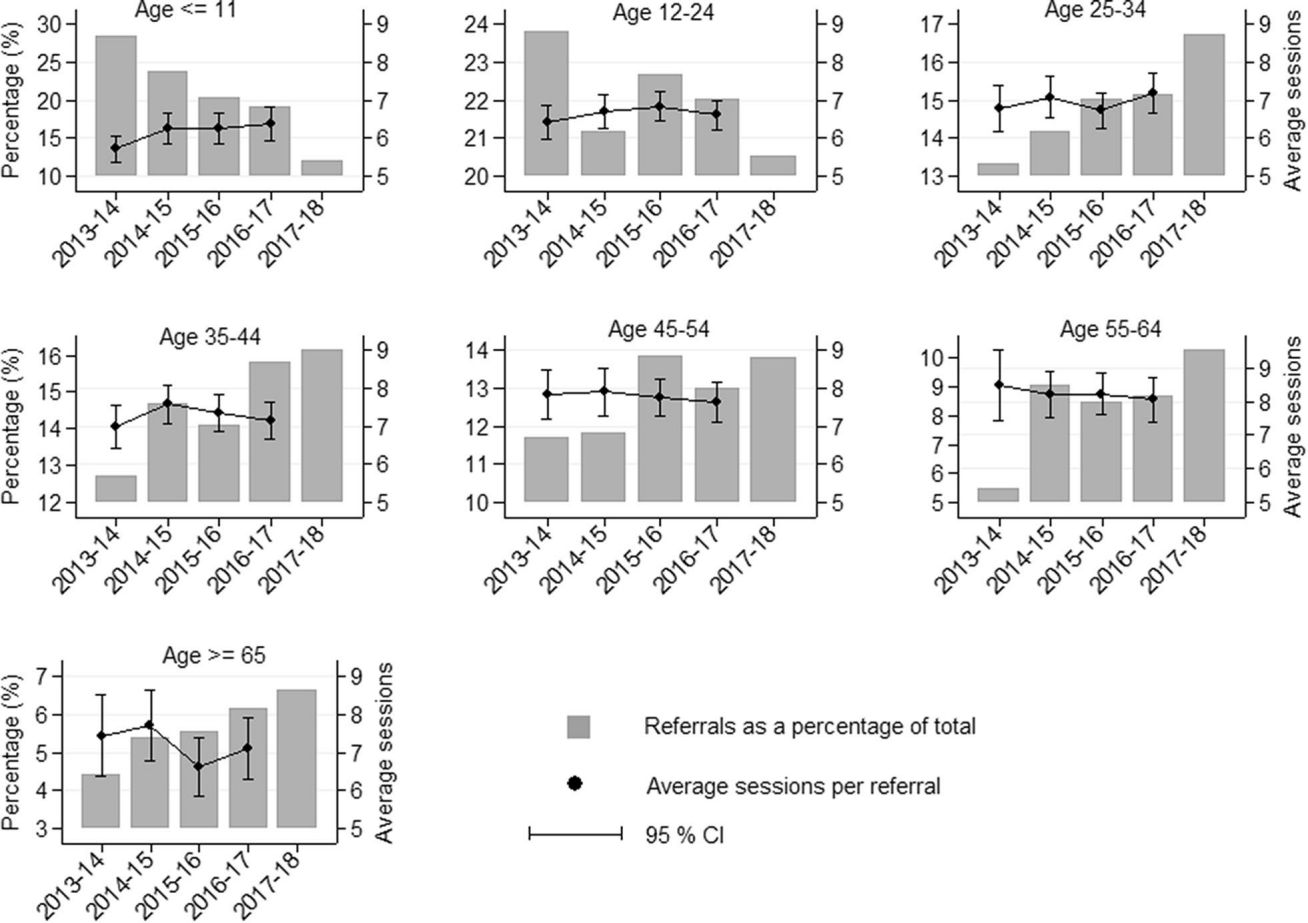
Referrals and sessions per referral for PMHC services by age categories, Western Sydney (Australia), 2013/2014–2017/2018. Primary axis range differ in each panel for a convenient visualisation; Average sessions per referral has not been calculated for 2017–2018 as not all referrals finished treatment sessions for 2018; *CI* confidence intervals

Younger age groups (0–24 years) were more readily referred than older age-groups, however the proportion of referrals among younger age-groups decreased over time from 2013–2014 to 2017–2018 particularly among those aged ≤ 11 years (Table 3 and Fig. 3). This was the case for referrals measured as a proportion of GP visits, or as a proportion of reported MBS items. Also, younger age-groups had high rates of non-attendance to the first service sessions and a lower mean number of sessions per referral for referrals resulted in services compared to older age groups (Table 2, Fig. 2).

**Table 3:** Age-specific frequency of PMHC referrals in Western Sydney (Australia) (2013–2014 to 2017–2018)

Age years	≤ 11	12–24	25–34	35–44	45–54	55–64	≥ 65
Population total = 1,027,211	177,843 (17.31)	173,286 (16.87)	176,645 (17.20)	157,832 (15.37)	121,880 (11.87)	101,806 (9.91)	117,919 (11.48)
GP visits (% of population)	3904 (1.96)	17,198 (8.29)	22,187 (11.20)	25,316 (14.65)	24,663 (16.59)	20,009 (16.39)	23,090 (16.20)
MBS items (% of population)	4923 (2.48)	15,375 (7.41)	15,762 (7.96)	16,148 (9.35)	13,754 (9.25)	9492 (7.78)	6876 (4.82)
2013–2014 referrals	623	520	291	277	255	120	96
% of GP visits^+^	17.04	3.21	1.36	1.10	1.03	0.59	0.40
% of MBS items	13.95	3.79	2.02	1.83	1.97	1.36	1.55
2014–2015 referrals	579	517	346	358	288	221	131
% of GP visits^+^	15.83	3.19	1.61	1.43	1.17	1.09	0.55
% of MBS items	12.21	3.51	2.28	2.27	2.17	2.39	1.99
Standardized % of GP visits	14.16	2.85	1.44	1.28	1.04	0.98	0.49
Standardized % of MBS items	10.92	3.14	2.04	2.03	1.94	2.14	1.78
2015–2016 referrals	578	647	429	402	395	242	158
% of GP visits	15.81	3.99	2.00	1.60	1.60	1.20	0.66
% of MBS items	11.23	4.02	2.60	2.42	2.76	2.50	2.20
Standardized % of GP visits	12.10	3.06	1.53	1.23	1.22	0.92	0.50
Standardized % of MBS items	8.59	3.08	1.99	1.85	2.11	1.91	1.68
2016–2017 referrals	606	695	478	499	410	274	195
% of GP visits	15.30	4.00	2.12	1.95	1.65	1.36	0.84
% of MBS items	11.36	4.09	2.81	2.93	2.83	2.69	2.58
Standardized % of GP visits	10.58	2.77	1.46	1.35	1.14	0.94	0.58
Standardized % of MBS items	7.85	2.83	1.94	2.02	1.96	1.86	1.79
2017–2018 referrals	647	1103	899	867	742	552	357
% of GP visits	15.80	6.12	3.99	3.44	3.03	2.80	1.62
% of MBS items*	12.13	6.49	5.28	5.09	5.13	5.42	4.73
Standardized % of GP visits	6.67	2.58	1.69	1.45	1.28	1.18	0.68
Standardized % of MBS items	5.12	2.74	2.23	2.15	2.17	2.29	2.00

Row percentages presented

PMHC services: Federal government funded mental health initiatives to support people otherwise have no or limited access to psychological treatments

GP visits: Any GP presentation related to anxiety, attention deficit hyperactivity disorder, bipolar disorders, depression, postnatal depression and schizophrenia

MBS items: Number of patients received Medicare rebates related to any mental health service received from a GP, Psychiatrist, Clinical psychologist or other allied health professionals

Standardised  %: Referral rates as a  % of GP visits and MBS rebates adjusted for number of referrals in 2013–2014

* 2016–2017 MBS rebates were used as the denominator

^+^2015–2016 GP visits were used as the denominator

## Discussion

This study investigated referral pathways and service utilization patterns of government funded mental health services in the region of Western Sydney (Australia), and is the first to describe service utilisation over the period (2005–2018) spanning the period prior to the transition to PHNs in 2016. There was an overall increase in referrals over the period (excluding 2010 and 2011), with exponential growth in referrals in 2017 and 2018 following the transition to PHNs in 2016. The reduction in referrals in 2011 likely reflects under-reporting during the period of transition of ATAPS services from divisions of general practice to Medicare locals [19]. Anxiety and depressive disorders were the predominant conditions among those referred, with 21% of referrals not taking up services, which is consistent with national ATAPS figures [20, 21], and also with population prevalence estimates of mental disorder [2]. Nearly sixty percent of referrals received were for females, slightly lower compared to recent national ATAPS figures [20, 21].

Waiting times for mental health treatments of greater than 1 month were observed among nearly a quarter of those referred and were higher among children aged ≤ 11 years. Longer waiting times among children may reflect that the majority presented with no suicidal risk, whereas those presenting to primary care with suicidal risk receive prompt access to the PMHC services [22].

Younger age groups also had higher non-attendance for first service session compared to older adults, and among those who attended service sessions, younger age groups attended a lower number of service sessions, on average, compared to older age groups. The higher number of treatment sessions among older age groups may be associated with increased mental health burden in terms of psychological distress [23], and may indicate higher burden of mental health conditions among older groups for those who referred to PMHC services compared to younger age groups. Higher proportions of non-attendance among young adults may reflect negative perceptions of mental health services, or a perception that mental health problems can be managed on their own or with the help of peers [7]. Additionally, it is also possible, especially among children, that parents or guardians are important facilitators of service utilisation, but may not consider referral to mental health services as sufficient [24].

Higher proportions of non-attendance among younger age groups could also reflect over referral to psychological treatments that are not required, particularly in earlier periods (Table 3 and Fig. 3). Age specific mental health encounters to primary care, as a percentage of total population, were higher among those aged > 24 years compared to those aged ≤ 24 years. However, the proportion of referrals to PMHC services was greater among those aged ≤ 24 years as a percentage of GP presentations and MBS rebates related to mental health conditions. This finding is consistent with previous research conducted in other settings for mental health services with similar characteristics [25, 26]. Pettit et al. suggested that older adults have lower access to the Improving Access to Psychological Services (IAPT) services compared to younger adults and further indicated that younger adults may be over referred to these services [25]. Prina et al. revealed that there was lower access to IAPT services among those aged over 65 than expected, despite they were benefited by these services [26]. In general, clients referred to key PMHC services in Western Sydney were younger in age compared to national ATAPS figures [21]. Additionally, marked downward trends in referral rates (as a percentage of GP visits, MBS rebates and total referrals) among those aged ≤ 11 years, and downward trends among those aged 12–24 years, in the most recent period also suggest over-referral to primary mental health care services in earlier periods of the study (Fig. 3).

While older age-groups had higher attendance rates to primary mental health service sessions, the number of referrals to these services was substantially lower than among younger age groups (despite high levels of primary care encounters being associated with mental health conditions in middle and older age-groups [27]). This may reflect that these older age-cohorts have a higher prevalence of comorbidity with other chronic conditions and physical disability and are less likely to be referred to mental health services [28–30].

## Limitations

There are a few of methodological limitations for consideration when interpreting the findings in the present study. Firstly, the current study does not include referral information from *headspace* services. Referrals from this service are not captured by the PHN, however this information may become available to PHNs in the near future. This will tend to underestimate the referral rates to PMHC services among youth those aged 12–24 years. Secondly, observed GP presentations relating to mental health conditions may differ from the actual number of GP presentations as individuals can visit more than one GPs. However, the GP presentation rates observed in the present study were consistent with estimates published by the Bettering the Evaluation and Care of Health study for 2015–2016 [27].

## Conclusion

Our study findings indicated that people experiencing mental health conditions have access to the PMHC services with an increasing trend in each year. Even though, this study found that younger people were more likely to be referred to the PMHC services than older people, but were less likely to attend services, which is consistent with findings in other countries. Since uptake is limited due to the capped funding, age specific disparities in access to the PMHC services should be eliminated, and further research needs to be designed to determine the causes to the inequalities in ensuring optimal referral pathways. Similarly, to assess whether these services are effective in reducing the incidents of hospital admissions related to mental health conditions, intentional self-harm, and suicides is important and designing data linkage studies are an utmost priority to better inform continuous system improvements.

## Data Availability

The PMHC MDS and ATAPS MDS are owned by the Australian Government Department of Health, and Western Sydney PHN has access to this data in its own catchment area.

## Abbreviations

PMHC: Primary Mental Health Care
ATAPS: Access to Allied Psychological Services
MDS: Minimum data set
PHN: Primary Health Network
IAPT: Improving access to psychological therapies
GP: General practitioner
IRSAD: Index of Relative Socioeconomic Advantage and Disadvantage
MBS: Medicare beneficiary schedule
SE: Standard error
IQR: Interquartile range
CI: Confidence interval
